# The 'antisocial' person: an insight in to biology, classification and current evidence on treatment

**DOI:** 10.1186/1744-859X-9-31

**Published:** 2010-07-06

**Authors:** Chaturaka Rodrigo, Senaka Rajapakse, Gamini Jayananda

**Affiliations:** 1 Mental Health Unit, Provincial General Hospital, Ratnapura, Sri Lanka; 2 Department of Clinical Medicine, Faculty of Medicine, University of Colombo, Sri Lanka

## Abstract

**Background:**

This review analyses and summarises the recent advances in understanding the neurobiology of violence and empathy, taxonomical issues on defining personality disorders characterised by disregard for social norms, evidence for efficacy of different treatment modalities and ethical implications in defining 'at-risk' individuals for preventive interventions.

**Methods:**

PubMed was searched with the keywords 'antisocial personality disorder', 'dissocial personality disorder' and 'psychopathy'. The search was limited to articles published in English over the last 10 years (1999 to 2009)

**Results:**

Both diagnostic manuals used in modern psychiatry, the *Diagnostic and Statistical Manual *published by the American Psychiatric Association and the *International Classification of Diseases *published by the World Health Organization, identify a personality disorder sharing similar traits. It is termed antisocial personality disorder in the diagnostic and statistical manual and dissocial personality disorder in the *International Classification of Diseases*. However, some authors query the ability of the existing manuals to identify a special category termed 'psychopathy', which in their opinion deserves special attention. On treatment-related issues, many psychological and behavioural therapies have shown success rates ranging from 25% to 62% in different cohorts. Multisystemic therapy and cognitive behaviour therapy have been proven efficacious in many trials. There is no substantial evidence for the efficacy of pharmacological therapy. Currently, the emphasis is on early identification and prevention of antisocial behaviour despite the ethical implications of defining at-risk children.

**Conclusions:**

Further research is needed in the areas of neuroendocrinological associations of violent behaviour, taxonomic existence of psychopathy and efficacy of treatment modalities.

## Introduction

The concept of a personality disorder with callousness and unemotionality plus disregard for social norms is well established in psychiatry [1]. Such people share a combination of traits that may include violence, aggression, callousness, lack of empathy and repeated acts of criminality against social norms. However, the classifications and definitions from this point onward are not clear.

Though such traits would have existed in human societies from time immemorial, identifying and classifying such behaviour has changed over time and continues to do so. In fact, the understanding of personality and its disorders were quite different in the early 19th century from their current context (which refers to a collection of traits that is expected to have a biological basis). Then, the term 'personality' was thought to be a more of a metaphysical issue. However, as the century progressed, measurement of personality in more objective terms, and hence the objective description of its disorders, gained popularity. A major turning point in this regard was the movement beyond the 'delusional definition of insanity' where the existence of disease of mind was accepted in absence of delusions [2]. For example, the theory of faculty psychology popularised in the 19th century considered the mind to have three separate faculties or bundles, namely intellect, emotion and volition. Concepts of disorders or 'insanities' of each component would later develop along the lines of schizophrenia, manic depressive illness and antisocial behaviour [3]. Despite these theories being challenged with time, they nevertheless helped to broaden the scope of classification of psychiatric illnesses to include the precursors of what is known as 'personality disorders' today.

Both diagnostic manuals used in modern psychiatry, the *Diagnostic and Statistical Manual*, currently on it's 4th edition (DSM-IV) published by the American Psychiatric Association (APA) [4] and the *International Classification of Diseases*, on it's 10th edition (ICD-10) published by the World Health Organization (WHO) [5], identify a personality disorder sharing similar traits (with certain disagreements). The DSM-IV classifies it as antisocial personality disorder (Axis II, Cluster B) while the corresponding diagnosis in ICD-10 is dissocial personality disorder. However, some authors argue that these criteria do not go far enough to define a third entity termed 'psychopathy' [6]. These blurred lines of classification, disagreement between mental health professionals, poor understanding of biological and non-biological factors (environmental) precipitating and maintaining such behaviour, add to the confusion.

This review aims to summarise and analyse the advances made in to understanding this phenomenon over the last decade in a scientific manner under several relevant topics: neurobiology of aggression, taxonomical efforts, advances in treatment and ethical issues in prevention.

## Methods

PubMed was searched with the keywords 'antisocial personality disorder', 'dissocial personality disorder' and 'psychopathy' using the software Endnote X2 (Thomson Reuters, Carlsbad, CA 92011, USA) to filter articles. The search was limited to articles published in English over the last 10 years (1999 to 2009). Bibliographies of cited literature were also searched. Relevant publications and epidemiological data were downloaded from websites of international agencies such as the WHO. All abstracts were read independently by the three reviewers, and relevant papers were identified for review of the full papers. The coding was performed by all reviewers independently, blinded to each other and entered in to broader categories relating to biology, taxonomy, treatment and prevention. Data sources included, reviews published in core clinical journals, cohort studies, interventional studies, case control studies, cross sectional analysis and epidemiological studies. The inter-reviewer agreement for data included in the final synthesis was 100%.

### The biology of empathy, callousness and aggression

Recent work on human and animal models has created an insight in to the biology of aggression and callousness. The influences of genetics, neurochemical signalling of the brain and the hormonal imbalances have been explored with some significant findings.

#### Neural connections

Empathy is defined as 'the ability to recognise and share another's emotional state' [7]. The neurocircuitry in experiencing empathy is thought to be organised in association with the limbic system. Many authors over the years have demonstrated the central role of the limbic system in forming and experiencing emotions including the mother-child bond, friendships and partner affiliations [8-10]. Recent studies have gone further to involve two structures closely related with the limbic system, the insula and the anterior cingulate cortex (ACC), to be central in experiencing and assessing emotions of self and others [10]. These findings are significant as they go beyond the neurobiology of emotions to explain the neurobiology of empathy.

The discovery of mirror neuron pathways (activation of motor areas of the brain when executing a task by self as well as while observing it being executed by another) was central in defining theories on neural pathways of empathy [11]. Firstly, this observation was extrapolated to hypothesise that the mirror neuron mechanism enables us to identify emotions such as fear, anger and disgust in others as we, ourselves, experience them [12,13]. Secondly, it was assumed that in the callous individuals, these pathways are abnormal compared to the 'normal population' [14-16].

On the first hypothesis, research has shown that the insula is activated when experiencing emotions (especially negative emotions such as pain and disgust) and when trying to imitate them [10,12,17]. It was also observed that similar activity occurred when observing similar emotions of other people. This was specifically demonstrated in relation to experiencing the pain of a loved one [18]. The second structure of concern, the ACC, has been shown to be closely linked with the autonomic nervous system. It is thought to coordinate an 'error detection mechanism' activated when something is 'wrong' [19]. It is also assumed to trigger an autonomic response in situations where such a response in warranted. The ACC also becomes activated in experiencing physical pain and social pain (social rejection) in self and others, which shows that it plays a role in experiencing the emotional component of pain [18,20]. Finally, a coordinated hyperactivity between the ACC and the insula has been demonstrated, which may explain a central role for these structures in experiencing emotions and empathy [19,21]. Several other areas such as the amygdala (part of the limbic system constantly activated in experiencing, expressing and learning emotions), orbitofrontal cortex (involved in controlling emotions, assessing positive/negative reinforcement and therefore involved in learning) and the ventromedial prefrontal cortex (activated in tasks involving moral decision making) are also thought to play a key role in experiencing empathy and maintaining socially acceptable behaviour [19,22,23]. In this regard, Kiehl *et al. *[24] describe the insula and ACC as constituting a 'paralimbic circuitry' (connecting limbic structures such as amygdala to cortical structures) which plays a central role in genesis of empathy.

A number of changes in these pathways have been described in antisocial or psychopathic individuals when compared to normal individuals. These include differences in activity during the performance of certain laboratory tasks related to experiencing and assessing emotions and decision making. Amygdala and orbitofrontal cortex (OFC) hypoactivity as well as ventromedial prefrontal cortex (vmPFC) dysfunction is shown to occur more frequently among those with callous and unemotional traits [14,16,25]. Similarly, individuals scoring higher for psychopathic traits have a reduced activity in the insular and ACC regions when exposed to tasks involving cooperation, emotion recognition and emotional memory. The reduced activity of limbic and paralimbic circuitry is believed to affect a person's ability to appreciate another's emotions (especially fear), to engage in appropriate prosocial behaviours (helping, comforting, altruism) and to avoid activities causing distress to others [24,26,27]. At the same time, the individual may have difficulty in processing his/her own emotions, assessing self-vulnerability and reducing behaviours that put him/herself at 'risk' [14].

#### Neurotransmitters and hormones

Recent findings indicate a role for serotonin, cortisol and testosterone in aggressive and antisocial behaviour [28]. Reduction of secretion of cortisol in response to stress (reactive), the strength of negative feedback on limbic and 'paralimbic' areas (feedback) and lesser cortisol levels at physiologically neutral states (basal), have all been shown to be correlated with socially disordered behaviour [10]. In a study of preschoolers, those who had more prosocial behaviour had higher basal serum cortisol levels [29]. Children with conduct disorders and aggressive traits had low basal cortisol levels [30]. Similarly, it was shown that callous and unemotional individuals had hyporesponsiveness in cortisol secretion in reaction to stressors [31]. The extent of aggression correlated with the degree of cortisol hyporesponsivity [32,33]. Given the fact that antisocial behaviour may be fashioned from childhood, such hormonal dysregulation and hyporesponsiveness may create permanent changes in cortical and subcortical connections, establishing a vicious cycle with time [10].

The 'known' physiological function of cortisol involves preparing the organism for adversity, creating sensitivity to fear and initiating withdrawal where appropriate. However, there may be many other unknown mechanisms of action of this hormone that mediate internal metabolism pathways and external interactions. For example, the mechanism by which the basal cortisol level is associated with aggression is unclear. However, these observations provide useful information where further research should be guided.

Dysregulation of the serotonergic neurotransmitter system is another area of interest. However, the evidence in this regard is not as strong as for cortisol. It is thought that serotonin helps to control aggression, impulsivity and disruption of this system results in less restraint. Indirect evidence for this hypothesis comes from reduction in aggressive and impulsive behaviour with selective serotonin reuptake inhibitors (SSRIs) in normal people [34]. However, attention must also be paid to recent criticism in attributing a presumed efficacy of SSRIs based on the neurotransmitter imbalance theory (see the section on Pharmacological treatment). In animal models, reduced activity in the serotonergic system is associated with increased attacks on non-vulnerable targets (offensive aggression). The predatory aggression toward vulnerable targets was unaffected [35]. There is also evidence that the serotonergic system closely interacts with the control of cortisol and testosterone secretion [28]. Disruption of the serotonin system is assumed to be partially responsible for cortisol hyporesponsiveness to stressors [36].

Under normal circumstances, testosterone is more associated with dominance and less with aggression. Despite animal studies showing an increased likelihood of aggression with high levels of endogenous or exogenous testosterone, the results from human studies are inconsistent [37]. This may imply that environmental and developmental factors such as learning and experience modulate the 'raw' biological effects. Complicating the picture further is the possible interactions of testosterone with neurotransmitters and their metabolism. For example, at low serotonin states, testosterone may promote aggression [28].

An interesting association between testosterone and a functional polymorphism of the monoamine oxidase A (*MAOA*) gene has been demonstrated by Sjoberg *et al. *[38]. The underlying hypothesis is that testosterone has a direct effect on transcription of the *MAOA *gene by acting on one of the promoters. However, stimulation of transcription is not as strong as that of glucocorticoids, which also bind to the promoter. When testosterone levels are high they may competitively inhibit glucocorticoid binding and result in less transcription of the gene. The product of the gene, monoamine oxidase A, breaks down a multitude of amines including serotonin. Using 95 male criminal alcoholics and 45 controls, Sjoberg and colleagues have shown that a combination of high level of cerebrospinal fluid testosterone and a low activity *MAOA *genotype were significantly predictive of antisocial behaviour and aggression in men.

In another experimental study, women participating in a bargaining game were administered sublingual testosterone. The group that received testosterone demonstrated fair bargaining behaviour and reduced conflicts compared to controls. However, people who thought that they received testosterone demonstrated more unfair bargaining. The authors interpret the findings to challenge the traditional view that testosterone is partly responsible for antisocial and aggressive behaviour. Instead, they suggest that it may help an individual to master a challenge and secure an advantage by demonstrating a situationally appropriate behaviour that may even be a prosocial one [39]. Still, two major limitations of the study are its experimental nature and the female only test population, which prevents the extrapolation of findings to real life events and the general population.

#### Genetics

The role of genetics in determining violence and aggressive behaviour has been examined recently. Continuing from the discussion above, in addition to the possible interaction with testosterone, the polymorphism of the *MAOA *gene is also assumed to have an interactive association with childhood adversity to predict aggression in males [40]. This observation has been repeated in several studies and offers an interesting example of a possible interaction of genetics with environmental factors [41,42].

Corley *et al. *[43] in analysing single nucleotide polymorphisms (SNPs) in a sample of adolescents with antisocial behaviour and drug dependence have reported significant gene-based associations for two genes, *CHRNA2 *and *OPRM1*, compared to controls. The first gene encodes for neuronal nicotinic receptor α-2 (associated with nicotinic dependence in schizophrenic families) [44] and the latter for the μ opioid receptor (implicated in many substance abuse behaviours previously) [45]. Similar findings for a genetic connection on a dual diagnosis of substance abuse and conduct disorder symptoms were reported by Stallings *et al. *[46]. They showed evidence of linkage for 9q34 chromosomal region when both vulnerability to drug dependency and conduct disorder symptoms were considered. There was also evidence for linkage to 17q12 region for conduct disorder symptoms alone. The evidence from twin and adoption studies show that both heredity and environment to have the same influence on antisocial behaviour [47]. However, a later analysis has shown that the influence of heredity is more in children with antisocial behaviour plus callous and unemotional traits compared to those without callousness [48,49].

In summary, aggression, unemotionality and callousness are not purely a result of environmental factors. Biology has an equal part to play. Evidence regarding the neurocircuitry of empathy and callousness has emerged in recent years. This system has a complex relationship with the neuroendocrine system via control and feedback mechanisms. A state of neuroendocrine imbalance (for example, less activity in paralimbic structures and hyporesponsiveness of the hypothalamic-pituitary-adrenal axis to stressful situations) contributes to callousness and unemotionality, which may self-perpetuate over time [10]. This brings forth the importance of early identification and treatment for a condition that was long considered untreatable. However, there are still many issues unanswered in this model; for example, what converts callousness to aggression in some and not in others?

### The 'antisocial', 'dissocial' and the 'psychopathic': the dilemma of classification

The antisocial personality disorder is a diagnosis made according to the DSM (from this point onwards DSM refers to the DSM-IV text revision (TR) published in 2000 unless otherwise specified) [4]. Allowing for some disparities, the corresponding diagnosis in ICD-10 is the dissocial personality disorder [5]. Both diagnostic criteria agree on several characteristics of the disorder they define: (a) lack of respect for social norms, obligations and irresponsibility; (b) reckless, irritable, violent and aggressive behaviour; and (c) lack of remorse or guilt.

However there are many traits that each classification has considered but not the other. Some important differences are specified below:

1. Lack of empathy (ICD-10 only).

2. Incapacity to maintain enduring relationships (ICD-10 only).

3. Repeated lying and conning others for personal benefit and pleasure (DSM-IV only).

4. Impulsivity and failure to plan ahead (DSM-IV only).

5. Reckless disregard for safety of self and others (DSM-IV only).

In addition, DSM-IV states that the individual must display a persistent disregard for rights of others since the age of 15, but at least be 18 years of age at time of diagnosis and also has a history of conduct disorder in childhood (not essential in ICD-10). In effect, DSM sets more stringent criteria for this diagnosis. However, lack of empathy, as shown previously is an important finding defined both biologically and behaviourally in a violent individual with a disordered personality (see above). Not including this as a definite diagnostic trait in DSM is notable.

In this context it is important to consider a third model for a corresponding/overlapping personality: psychopathy. Diagnosing psychopathy as a separate entity has created intense debate [50-53]. Currently, neither APA nor WHO recognise psychopathy as a separate entity, but something synonymous for the corresponding personality disorders defined in their criteria [4,5]. The concept of psychopathy as a separate diagnostic entity was promoted by Hare *et al.*, who developed the Psychopathy Checklist - Revised (PCL-R) to diagnose it [54]. The psychopath is said to have a combination of violent, aggressive and callous traits plus a narcissistic, superficially charming, manipulative, emotionally shallow nature with a background of criminality and social deviance [55]. The argument is that while the antisocial and dissocial disorders concentrate on violent, impulsive and aggressive behaviour, psychopaths may represent a subset that has superficial charm and manipulativeness with pathological lack of concern for others. Their existence is characterised by more practical terms of measurement such as violent crimes, criminal recidivism and misbehaviour even during imprisonment [56,57].

The original PCL-R scored 20 items, many of which were grouped into two clusters termed factors 1 and 2. Each item is scored on an ordinal scale of 0, 1 or 2 (maximum score of 40). The score is determined on a detailed assessment with semistructured interviews, details of records and information from other relevant sources. The scoring has to be performed by experienced clinicians well versed in the scoring manual given the ethical and legal implications of a positive diagnosis. Various cut-offs are used to define psychopathy depending on the setting and context (whether for judicial or research purposes) [58].

Factor 1 traits are more towards aggressive narcissism (superficial charm, emotional shallowness, lack of responsibility, callousness, lack of empathy, grandiose self-worth) while the factor 2 traits are more towards a socially deviant lifestyle (juvenile delinquency, early behavioural problems, poor self-control, impulsivity, lack of long-term goals) [54]. To be defined psychopathic, an individual has to score high on both factors. Instead of the two-factor model of PCL-R, it is also proposed that the construct of psychopathy can be better explained by categorising the same items under a three-factor (interpersonal, affective, behavioural/lifestyle) or four-factor model (interpersonal, affective, lifestyle and antisocial) [59,60].

There are two derivatives of the original PCL-R that are also used to assess psychopathy. The PCL:SV (short version) is a shorter 12-item scoring system (also scored on a 3-step ordinal scale) that is used to screen for psychopathy in forensic and civil psychiatric patients. The PCL:YV (youth version) is a 20-item scale that is a modified form of PCL-R to assess adolescents and young offenders. Given the implications of labelling young individuals as psychopathic, this scale is not intended as a diagnostic tool [58].

There seems to be an overlap of the items of factors in PCL-R with the more 'official' diagnoses in the diagnostic manuals (considering the two-factor model, it is observed that DSM-IV criteria for antisocial personality disorder (ASPD) falls more towards factor 2 traits, while dissocial personality disorder of ICD-10 also includes some factor 1 traits). This overlap of some traits and the exclusion of others in these diagnostic criteria has led to a debate as to whether each of these 'diagnoses' are separate entities (categorical) or subsections of a continuum of personality disorders (dimensional) [50,52].

Several studies provide evidence for the existence and the categorical nature of psychopathy. Earlier studies by Harris *et al. *[61] in Canada supported the idea that a taxon can be identified for psychopathy based on the application of PCL-R to mentally disordered offenders. However the evidence for a taxon existed for factor 2 traits (which correlate more with ASPD) rather than the factor 1 traits of PCL-R. Furthermore, many queries regarding the methodology and results of this study were raised later [55]. Warren *et al. *[62] have assessed the similarities and dissimilarities of individuals fulfilling diagnostic requirement for ASPD and psychopathy (using 137 female incarcerated offenders). ASPD was characterised by aggressive, impulsive behaviour plus a greater association with cluster A personality disorders. Psychopathy was associated with remorselessness, previous imprisonments and criminality. However, both were similar in disregarding of social norms and deception. The authors concluded that the two disorders are not synonymous and different treatment strategies may be required to tackle each diagnosis. The generalisability of these findings is doubtful given the small sample size and the gender bias of the sample. Cunliffe and Gacono [63] applied PCL-R to 45 incarcerated female offenders diagnosed with ASPD. The psychopaths and non-psychopaths were then compared with Rorschach measures with regard to self-perception, interpersonal relatedness and reality testing (social perception/perceptual accuracy). Those having a dual diagnosis of ASPD and psychopathy demonstrated considerable disturbances in these measures, distinguishing them from ASPD individuals without psychopathy.

There is also controversy on the agreement between different diagnostic criteria for the same disorder. Rutherford *et al. *[6] applied 5 diagnostic criteria (Feighner criteria, Research Diagnostic Criteria (RDC), DSM-III, DSM-III-R, and DSM-IV) to a single sample of 137 women to diagnose ASPD. The diagnostic rates for ASPD varied from 11% (RDC) to 76% (Feighner criteria). In addition, after applying the PCL-R to diagnose psychopathy, considerable overlap existed between this diagnosis and ASPD when different criteria were used. The authors concluded that psychopathy and ASPD are not synonymous terms.

With regard to evidence to the contrary, Marcus *et al. *demonstrated (on a sample of prison inmates) that there is no evidence for a categorical structure in psychopathy [52]. However in contrast to Harris *et al*., they used the Psychopathic Personality Inventory (PPI) instead of the PCL-R to assess core psychopathic personality dimensions, which limits a direct comparison. Later, Edens *et al. *[55] using the PCL-R on a sample of prison inmates still failed to demonstrate the categorical nature of psychopathy. In addition, Marcus *et al. *[64] also argue that ASPD is a dimensional entity best assessed in a continuum rather than as a categorical diagnosis. Their findings are based on applying the Structured Clinical Interview for DSM-IV axis II Personality Disorders (SCID II) and the Personality Diagnostic Questionnaire 4 (PDQ-4) ASPD scale to 1,146 male offenders.

Taxometric analysis of personality disorders is a vast area on its own. The brief description above is included to highlight the diagnostic differences, controversies and discrepancies between assessment methods. It is fair to summarise that there is considerable non-agreement between different diagnostic systems identifying a personality disorder characterised by gross disregard for social norms and remorselessness. In fact, the validity of a direct comparison of studies using various diagnostic criteria is questionable as the populations diagnosed are different (see Rutherford *et al. *[6]).

While the categorical or dimensional nature of psychopathy is debated, some authors have shown that ASPD itself may be a dimensional diagnosis [64]. The 'dimensional' nature infers to two scientifically important issues [55,64]: (1) it exists in a continuum in many population subgroups, and (2) it is more likely to have a multifactorial aetiology. These attributes have a direct impact on treatment and preventive strategies.

### Treatment, outcome and therapeutic pessimism: is it permanent 'brain damage'?

Treatment of antisocial personality disorder and psychopathy is no longer viewed with pessimism [65]. The traditional method of punishment for socially deviant behaviour by incarceration is not considered effective in preventing recidivism [66]. The more positively structured interventions (rehabilitative rather than punitive) can be either family-based (multisystemic therapy, functional family therapy) or in a residential setting (therapeutic community). Though the first option is considered to be better, sometimes the law requires residential placement [67]. Another interesting theory on treatment is the iatrogenic reinforcement of criminal behaviour. Some argue that treatment approaches themselves may promote criminal behaviour (repeated discussions in group therapy, association with deviant peers, sharing of experiences). However there is no evidence to confirm this hypothesis [68].

Though time consuming, intense psychotherapeutic programmes have shown benefit [69]. Rather than categorising ASPD as untreatable, spending more time with patients is shown to increase entry in to a treatment programme [70]. Social workers or case managers play an important role in this regard. The positive impact of psychotherapy in psychopathy was assessed in a meta-analysis by Salekin *et al. *[65]. They analysed 42 interventional studies for individuals classified as psychopathic (unfortunately, the studies used different criteria to diagnose psychopathy: Cleckley, Hare, Craft, Partridge, Gough, and several other criteria). Despite the method used to classify psychopathy, patients in treatment groups improved with therapy compared to controls. Overall, 60% showed improvement even after dropping the individual case studies and this improvement was significantly better than the control groups (*P *< 0.01). Cognitive behavioural therapy and psychoanalytic psychotherapy were the most successful treatment modalities (with 62% and 59% of clients improving, respectively). The therapeutic community approach was the least successful with only a 25% success rate. In control groups, without any formal intervention, 19.8% improved over time. It was also demonstrated that a younger age and a longer duration of therapy had a positive correlation with better outcome. This meta-analysis included studies conducted from the 1940 s to 1990. The following sections analyse evidence of benefit with each interventional modality from more recent studies.

#### Cognitive behaviour therapy (CBT)

The fundamental principal in CBT is to alter the thinking process to induce a behavioural change [71,72]. It is an established practice in treatment of ASPD, psychopathy and currently included as a treatment option for ASPD in the UK National Institute for Clinical and Health Excellence (NICE) guidelines [73,74]. In the meta-analysis quoted above, CBT was the most successful intervention strategy for psychopathy [65]. A Cochrane review of residential treatment programmes with CBT by Armelius *et al. *[66] confirms the beneficial impact of CBT for antisocial youth. In the overall analysis, there was an improvement of outcome measures (police or court reports, self-reports of violence, readmission to a residential facility and any other official evidence of an offence) in the CBT-treated group compared to the control group at 12 months of follow-up (odds ratio (OR) 0.69). At this point there was a reduction in recidivism of 10% for the CBT group. The impact of CBT was more than any other alternative psychotherapeutic intervention (attention control, stress management). However, no difference between the other groups and the CBT-treated group was observed at 6 and 24 months. This may be attributable to a too short follow-up time to elicit positive outcomes (at 6 months) and the absence of a long lasting impact of CBT (at 24 months). In a more recent randomised control study, Davidson *et al. *[75] assessed the outcome of CBT in a group of males (n = 52) with ASPD in a community setting (as opposed to residential patients). There was no difference in outcome in the CBT group compared to standard treatment group at 12 months. However, improvements were seen in both groups. In another CBT-based treatment programme for sex offenders, 85% completed treatment. The dropout rate was higher in individuals diagnosed as psychopathic (PCL-R), though 75% of them also completed the treatment. On a follow-up of over 10 years (on average), 54.5% were charged with a new crime (sexual or violent) but the highest rates of recidivism were among the psychopathic dropouts [76]. Kunz *et al. *[72] also followed-up a ASPD cohort treated with CBT. After a 4-year follow-up, 35% were considered stable (without significant behavioural problems, rearrests or rehospitalisations).

While the efficacy of CBT has been established, it is also important to note some criticisms regarding it. On a more ethical and a philosophical note, it can be argued that CBT is a form of mind control and the therapist's viewpoints are imposed on their client. Some argue that it does not address the core issues of mental instability and restricts therapy to goals and targets set by therapists. On a more pragmatic scale, CBT is time consuming and needs trained staff. It cannot be successfully applied to clients with subnormal intelligence [77]. Furthermore, engaging and making behavioural changes in antisocial clients can be a demanding task for a therapist.

#### Multisystemic therapy (MST)

MST is delivered in a family-based setting by a dedicated full time staff with an emphasis on a flexible and individualised treatment schedule. Its main focus is children and adolescents with socially deviant behaviour [69,78]. The core principles of MST include identifying problem behaviours in the broader systemic context (self, family, environment), using the strengths in each context for positive change, promoting responsible behaviour in the family, targeting specific problems with time limits and attempting to address many flaws in different systems that contribute to the problem behaviour (for example, family, school, neighbourhood, government authorities). Such interventions are a collaborative effort of therapists, the patient's family and the patient. The schedule is tailored according to the developmental needs of the patient (child or adolescent). The final aim is the long-term empowerment of the patient and care givers to maintain the positive behaviour [79]. The first assessment on MST was published in 1986 by Henggeler *et al. *[80] (n = 80) and showed that MST reduced behavioural problems, deviant peer association and improved family communications compared to standard therapy in juvenile offenders. Subsequently it was demonstrated that in addition to the above benefits, MST clients also had significantly lower rates of recidivism and rearrest [81]. Bourdin *et al. *[82] in a randomised clinical trial (n = 200) compared MST versus individual therapy and concluded that MST completers had significantly lower rearrests and recidivism (significantly less rearrests for sexual offences, substance use related offences and violent aggression). A meta analysis on trial data (11 studies with 708 participants) of MST shows improvement of patient and family functioning compared to 70% of others treated differently [83]. It also shows that better results are dependent on the therapists as well (graduate trainees performing better than community therapists). While MST has demonstrated positive effects on improving family relations and reducing antisocial behaviour, it is targeted more towards juvenile offenders with family support. It is a time-consuming exercise and requires a high degree of personal attention from therapists. The difficulty in applying MST for adults and in situations without family support plus the scarcity of trained therapists limits its use in treatment.

#### Other psychological and behavioural treatment options

Many different psychological and behavioural therapies have been tried in people with antisocial behaviour. Some are targeted at individuals alone while others involve the family and the immediate environment of the client. The role of psychoanalytical psychotherapy was shown to have a positive effect on psychopathy in earlier studies but there is no recent evidence in this regard [65]. Bateman *et al. *[84] describes the use of mentalisation-based treatment to counter ASPD. Their argument is that ASPD individuals do not have a sound mentalisation process (the ability to gauge and interpret the purposefulness of actions of self and others, based on intentional mental states such as needs and beliefs). In this sense, they are more likely to misinterpret the behaviours of others. Their incapacity is protected by rather rigid, inflexible conceptualisations. When they are challenged, the person may resort to violence and aggression to control the situation. Since the ASPD individual lacks empathy, it is thought that mentalising about one's own mental state at times of stress will be more useful than taking examples focused on others. However this method of therapy has not been assessed by a controlled clinical trial.

The therapeutic community approach is another option for rehabilitating offenders with personality disorders in a community setting. In this situation the therapists and other service providers live with the clients in a 'community' continuing the rehabilitation process. However, Salekin *et al. *(see above) in their meta-analysis concluded that it is much less useful than CBT or psychoanalytic psychotherapy (25% success rate vs 62% with CBT). However, it is notable that the therapeutic communities had a larger number of clients compared to the limited numbers in a CBT program. In this instance, 372 in the therapeutic communities versus 246 in CBT groups. With this taken in to account, patients on CBT were only 1.6 times more likely to improve than those in therapeutic communities. However, other confounding factors such as differences in measures of outcome, therapeutic exposure and techniques of therapy would have affected the efficacy rates. In a more recent analysis, Blumenthal *et al. *[85] assessed the outcome of high-risk offenders (sexual and violent crime offenders) rehabilitated in a specialist hostel. Of 80 offenders admitted, 50 (63%) left the facility within 2 years after successful rehabilitation. Higher scores on PCL-R and being arrested for violence were poor prognostic indicators. In another study on therapeutic communities, patients diagnosed with ASPD (according to the Milton Clinical Multiaxial Inventory (MCMI II)) and other offenders were randomised to two groups (n = 187 and 88, respectively). In all, 42% of the total sample completed therapy and there was no difference between the ASPD group and others, indicating that such a diagnosis is not an indicator of therapeutic failure [86].

Family-based treatment strategies are predominantly aimed at juvenile offenders and children at risk of developing ASPD in adulthood (for example, children with conduct disorders) based on the hypothesis that the family dynamics are partly responsible for the personality attributes [87]. Rehabilitation within the family unit is considered more feasible, practical and sustainable. NICE guidelines suggest treatment strategies such as parent training, brief strategic family therapy (supporting the family, identifying and correcting maladaptive family behaviours) or functional family therapy (problem solving, behavioural change with application of such change in social functioning) in managing adolescents with ASPD or at risk of ASPD [74]. If the patient's behaviour is problematic and it is likely that foster care/residential treatment is necessary, multisystemic therapy is recommended. However, the evidence on efficacy on these methods is scarce and the guideline itself points out the need for randomised trials to compare family-based strategies with other methods such as multisystemic therapy.

Contingency management (CM) is a behavioural therapy that uses rewards (or rarely, punishments) to induce a behavioural change. It is used in substance abuse treatment and may involve a token economy system (vouchers for good behaviour) or a level system where those graduating to a specific level are entitled to specific benefits that are not available to the others at a lower level. Messina *et al. *[88] assessed the efficacy of CBT and CM in a group of cocaine-dependent (with and without ASPD) patients. The patients (n = 120) were randomly assigned to four treatment groups (CBT, CM, CBT + CM, methadone maintenance). Overall, patients with ASPD responded better (abstaining from drug use) than those without ASPD. The CM group had the overall best response rate as assessed by cocaine-negative urine samples. ASPD patients in the CM group performed significantly better than their ASPD-negative counterparts (*P *< 0.05). The traditional method of methadone maintenance had the least efficacy and therapeutic failure was significantly more in ASPD individuals.

#### Pharmacological therapy

Pharmacological therapy for ASPD *per se *is considered ineffective and not recommended in NICE guidelines [74]. However, it has a place in treating concurrent psychiatric disorders such as depression and anxiety. Given the biological associations of antisocial behaviour (neurotransmitter and hormonal imbalances) the role of pharmacological agents cannot be completely ruled out. An area of interest is the use of selective serotonin reuptake inhibitors (SSRIs). It has been shown that aggression may be linked to dysfunction of the serotonergic nervous system and SSRIs are effective in controlling emotional aggression in personality disorders. However, it has not been shown to be effective in controlling aggression in repeat offenders [34]. Paroxetine (an SSRI), has been shown to improve cooperative behaviour in normal people, but this effect has not been demonstrated in populations with antisocial behaviour [74]. Hirose [89] reported a case of a patient with ASPD treated with risperidone. His aggression was controlled with risperidone but it is not mentioned whether he received concurrent psychotherapy. The author attributes the 5HT_2 _(serotonin receptor) antagonism of risperidone to its therapeutic effect. The observations of this individual case study have not been confirmed by others.

The inefficacy of pharmacological treatment may be approached by a different hypothesis. This argument stems from the work by Moncrieff *et al. *on developing an alternative hypothesis regarding the efficacy (or rather the lack of it) of antidepressants [90-92]. They propose to use a drug-based approach to understand the effect of antidepressants rather than the traditional disease-based approach. To clarify this further, the diseased-based approach assumes that the therapeutic efficacy of drugs emanate from their ability to alter the disease pathology (for example, SSRIs increase the serotonin concentrations that act on synaptic receptors, hence offsetting the serotonin 'depletion' that led to depression). The drug-based model proposes that the therapeutic effect of drugs is coincidental and dependent on social context. Instead of acting on the presumed biochemical model of disease causation, the drugs may create a different biochemical environment that may coincidentally relieve symptoms. It goes further to state that, in such a situation, the effect may not differ between placebo and the drug. To support this view authors cite the questionable efficacy of antidepressants when prescribed over a longer timescale, the ability of other non-antidepressants to improve scoring on depression scales via their sedative/stimulating effects, and the conflicting evidence from randomised clinical trials on the efficacy of antidepressants.

Applying this concept to ASPD, the following argument can be elicited. Though many presumed biochemical associations (though evidence is limited and conflicting at times) have been described (neurotransmitter and hormonal disturbances), the observed efficacy of drugs is far less than expected from a disease-based model. This can be explained in two ways:

1. Shifting the focus to the drug-based model, it can be argued that the neurochemical alterations induced by therapy have no or minimal impact on the pathology of ASPD.

2. If accepting a disease-based model, it may be that the drugs are ineffective because the assumed model of pathology is erroneous.

The sporadic efficacy of drugs on occasional case reports may be attributed to various other issues such as the social context and sedative or anxiolytic effects that lead to temporary abatement of symptoms. The hypothesis of Moncrieff *et al. *states that the antidepressants are unlikely to have a significant impact when prescribed long term. Following this, it can also be argued that the long-term 'visible' morbidity and social disturbance of ASPD is far more compared to depression. Therefore the lack of efficacy of pharmacological therapy or 'incurability' would be far more obvious in ASPD than in depression.

However, it needs to be stressed that given the paucity of evidence for a biochemical model for symptoms in ASPD, it is not possible to either accept or reject any of the hypotheses presented above.

In summary, the therapeutic pessimism on ASPD and psychopathy is unwarranted. Many psychological and behavioural treatments have shown beneficial effects ranging from 25% to 62% in different cohorts. Multisystemic therapy and cognitive behaviour therapy have proven their efficacy in many trials. The evidence for therapeutic community approach is conflicting. The family-based treatment strategies for juvenile patients are recommended but its efficacy is not proven. Contingency management is shown to be a good approach to ASPD patients with substance abuse and this has to be explored further. There is no substantial evidence base for the use of pharmacological therapy other than to treat concurrent psychiatric disorders.

### 'Prevention' of antisocial behaviour: the ethical dilemma

A diagnosis of psychopathy, ASPD or dissocial personality disorder is associated with stigma. At the same time, such a diagnosis has a significant impact on limiting educational, vocational, social and other opportunities in life. Based on this premise, great care has to be taken in making a formal diagnosis. Recent research has focused on identifying at-risk individuals for antisocial/dissocial personality disorder. The positive impact of such an exercise is that it enables early intervention and stalls the progression to full-scale personality disorder that causes considerable personal and social distress. At the same time, preventing such personality disorders may be cost effective than rehabilitating the personality disordered adults. However, identifying such 'at-risk' individuals poses an ethical dilemma. Given the stigma, it may not be fair to label someone as 'at risk' of ASPD/psychopathy when the positive predictive values of criteria themselves are questioned. Identification of 'at-risk' individuals should ideally take place at an early age and making such a categorisation in children may be an infringement of their rights. However, the current opinion favours the identification and implementation of early intervention strategies for the 'at-risk' children [74].

The conventional risk factors of antisocial behaviour and associated personality disorders in adulthood include behavioural problems, attention deficit hyperactivity disorder and conduct disorder in childhood. In DSM-IV, childhood conduct disorder is an essential criterion to diagnose ASPD in adulthood. It has been shown that at least one-third of hyperactive children develop conduct disorder in late childhood and about half of this subgroup is diagnosed with ASPD in adulthood [93]. However, the treatment for attention deficit hyperactivity disorder (ADHD) and conduct disorder itself is not satisfactory and therefore identification and intervention at an earlier stage was considered necessary. The NICE guidelines currently recommend interventions for even preschool children considered 'at risk'. Naturally this calls for a redefining of markers for screening. In this regard, the focus has shifted from the child to the child's environment. While child-related factors such as callousness are still important, more family-related markers such as delinquent siblings, young parents, history of residential care, convictions by criminal justice system or mental illness of parents are given more emphasis [74]. Given these criteria, it is understood that a significant number of children will be considered at risk and a significant number of parents will be deemed at risk of raising such a child. Considering the ethical implications, lack of substantiate evidence on efficacy of interventions and the uncertainty of diagnosis itself, some authors have called for a wider public discussion on this issue [73].

### Limitations

This review was limited to articles published in English within the last decade. While attempts were made to search related literature as well, it is possible that important studies published in other languages and outside the search limits were missed.

## Conclusions

Aggression, lack of emotions and callousness are a combined consequence of genetics, neurotransmitter/hormonal imbalance and environmental factors. Many recent advances have been made in to understanding the complex interrelations of the neurocircuitry underpinning empathy and emotions. There is intense debate as to whether a separate diagnosis of psychopathy exists, but neither antisocial personality disorder as defined in DSM-IV nor its corresponding diagnosis in ICD-10, dissocial personality disorder, identify psychopathy as a separate diagnosis. A major problem for scholars summarising evidence on this type of personality disorder is the differences in various diagnostic criteria. The populations diagnosed with these criteria sometimes differ considerably, so a direct comparison of results is difficult. On treatment-related issues, many psychological and behavioural therapies have shown success rates ranging from 25% to 62% in different cohorts. Multisystemic therapy and cognitive behaviour therapy have been proven efficacious in many trials. Given the social and personal costs involved, some authorities such as the UK National Health Service (NHS) recommend identification of at-risk children and intervention at an early age. This raises several ethical issues that need to be addressed by a wider public discussion.

Further exploration of the inter-relationship between neurocircuitry, neurotransmitters and hormones regarding empathy and violence, consensus among different professional bodies on a uniform criteria to diagnose antisocial personality disorder with clarification of taxonomic existence of 'psychopathy', randomised, controlled clinical trials to compare the treatment efficacy of therapeutic communities, family-based management strategies and contingency management, and randomised controlled trials to assess the efficacy of early interventional therapy for 'at-risk' children are identified as areas for further research.

## Author information

CR (MBBS) is a medical officer of mental health attached to the psychiatry unit of Provincial General Hospital, Ratnapura, Sri Lanka. GJ (MBBS, MD (psych)) is the consultant psychiatrist of the unit. SR (MBBS, MD, MRCP) is the head and senior lecturer of the Department of Clinical Medicine, Faculty of Medicine, University of Colombo, Sri Lanka.

## Competing interests

The authors declare that they have no competing interests.

## Authors' contributions

All authors participated in designing, article search, information coding and writing of the manuscript. All authors read and approved the final manuscript.
